# Promotion of recovery from Traumatic Brain Injury (TBI) by Granulocyte Colony-Stimulating Factor (G-CSF) treatment requires cannabinoid receptor type 2 activity

**DOI:** 10.1186/s42238-025-00305-8

**Published:** 2025-08-18

**Authors:** Shijie Song, Bangmei Wang, Xiaoyuan Kong, Niketa Patel, Juan Sanchez-Ramos, Mark S. Kindy

**Affiliations:** 1 Research Service, James Haley VA Medical Center, Tampa, FL USA; 2 Department of Neurology, Morsani College of Medicine, University of South Florida, Tampa, FL USA; 3 Department of Molecular Medicine, Morsani College of Medicine, University of South Florida, Tampa, FL USA; 4 Department of Pharmaceutical Sciences, Taneja College of Pharmacy, University of South Florida, Tampa, FL USA; 5 James Haley VA Medical Center and Department of Neurology, Morsani College of Medicine, University of South Florida, Tampa, FL 33612 USA; 6 James Haley VA Medical Center and Department of Pharmaceutical Sciences, Taneja College of Pharmacy, University of South Florida, Tampa, FL 33612 USA

**Keywords:** Controlled cortical impact (CCI), Traumatic brain injury (TBI), Brain repair, Granulocyte-colony stimulating factor (G-CSF), Endogenous cannabinoid system (eCBs), Cannabinoid receptors type 1 and type 2

## Abstract

Granulocyte colony-stimulating factor (G-CSF) has the capacity to enhance brain repair following various injuries to brain. G-CSF treatment after TBI in rodents has been reported to promote brain repair, hippocampal neurogenesis, and behavioral recovery. Delta9-THC treatment also enhances brain repair after TBI, and triggers upregulation of G-CSF in brain, raising the question as to whether G-CSF mediates recovery via the eCBs. A recent report revealed that pharmacological blockade of CB1 and CB2 receptors did not impede recovery from CCI. Given that pharmacological blockade of receptors has limitations, studies were conducted in mice with ablated or “knocked out” CB2R (CB2R KO mice). The hypothesis to be tested is that G-CSF enhancement of brain repair does not require activity of CB2 receptors.

**Results and discussion** G-CSF administration for 3 days after CCI did not enhance recovery of balance and coordination measured on the rotometer in CB2R KO mice, unlike the beneficial effects of G-CSF treatment observed in normal control mice. Even before CCI, the CB2R mice were markedly impaired on the rotometer, suggesting that activity of CB2R is important for normal function of neural networks that mediate balance and coordination. Expression of CB2R was increased by G-CSF treatment in normal mice 3 days after CCI but not in CB2R KO mice. Interestingly, the CB1R in the CB2R KO mice was upregulated by G-CSF treatment indicating that “knocking-out” or lowering expression of CB2R did not impact expression of CB1R. Expression of the neurotrophic factors BDNF and GDNF did not change with G-CSF treatment in CB2R KO mice. Levels of the endogenous cannabinoid ligand, 2-AG, were shown to be increased by G-CSF treatment in the CB2R KO mice, but upregulation of 2-AG does not appear to promote recovery of balance and coordination. Additional studies will be required of other components of the eCBs.

**Conclusion** The hypothesis that G-CSF enhancement of brain repair does not require activity of CB2 receptors is disproven by data in this report. The eCBs, in particular activity of the CB2R, is critical for G-CSF promotion of recovery of balance and coordination impaired by CCI.

## Introduction

Traumatic brain injury (TBI) has been defined as an insult to the brain caused by an external physical force that may produce a diminished or altered state of consciousness and that results in an impairment of cognitive abilities or physical functioning (Cernak 2005). The pathophysiology of TBI is a complex process encompassing overlapping phases of injury and recovery. Most studies focus on the “secondary injury” which comprises physiological, neuroinflammatory, and biochemical processes triggered by the primary insult (Cernak 2005). The “secondary injury” develops over hours to days. Chronic post- traumatic neurologic disability is a consequence of these secondary injuries. The full extent of neurological disability depends on mitigating effects of the reparative and regenerative responses triggered by the trauma.

There is a need for pharmacotherapeutic agents to promote recovery from TBI. G-CSF is a hematopoietic growth factor (HGF) that plays a crucial role in regulating the production of white blood cells (specifically granulocytes) in the bone marrow. It is commonly used in clinical settings to treat conditions associated with low white blood cell counts (leukopenia) caused by factors like chemotherapy or bone marrow disorders. G-CSF stimulates the proliferation and differentiation of precursor cells in the bone marrow, leading to an increase in the production of granulocytes, which are important components of the immune system. However, G-CSF has also been investigated in preclinical and experimental settings for its potential therapeutic effects in other contexts, such as stroke and traumatic brain injury (TBI) in rodent models (Schabitz et al. 2003; Six et al. 2003; Shyu et al. 2006; Solaroglu et al. 2006). A course of intra-peritoneal G-CSF administration (via osmotic minipump) initiated concomitantly with TBI in a rat model resulted in significantly better motor function recovery than in the control group (Yang et al. 2010).

These findings have been extended to a longer follow up period (2 weeks after mild CCI) in a different species (mice) treated with a lower doses of G-CSF (100 mg/kg for 3 days) after the injury (Song et al. 2016). Treatment with GCSF resulted in more rapid recovery from a spatial learning deficit than vehicle-treated mice. Enhanced recovery in the spatial learning task was associated with attenuation of the subacute neuroinflammatory response to the injury (Song et al. 2016). Interestingly, the GCSF treated mice showed increased hippocampal neurogenesis as well as increased microgliosis and astrocytosis.

Among the cellular, neurochemical and physiological changes triggered by TBI, alterations in the endocannabinoid system (eCBs) appear to play a significant role in modulating brain repair. It has been known for some time that eCBs participates in the brain’s complex cascade of responses to injury by decreasing vasoconstriction, gliosis, excitotoxicity and neuroinflammation (Song et al. 2016). The basic components of the eCBs include ligands, N-arachidonoyl ethanolamine (AEA) and 2-arachidonoyl-glycerol (2-AG), receptors (CB1, CB2), transporters, and enzymes, which are responsible for the synthesis and degradation of these lipid mediators (Piomelli 2003). Studies have shown that CB1 and CB2 receptors and their endogenous ligands can modulate cellular and molecular pathways of TBI pathology including cell death, excitotoxicity, neuroinflammation, cerebrovascular breakdown, and cell structure and remodeling (Schurman and Lichtman 2017; Zhu et al. 2021). TBI-induced behavioral deficits, such as learning and memory, neurological motor deficits, post-traumatic seizures, and anxiety also react to manipulations of the endocannabinoid system (Chen 2023; Song et al. 2022a). TBI produced by a weight drop model in mice resulted in decreased expression of brain cannabinoid-1 receptors (CB1) and increased expression of cannabinoid-2 receptors (CB2R) (Shohami et al. 2011; Lopez-Rodriguez et al. 2015). This finding was replicated following CCI in mice, which exhibited a dramatic upregulation of CB2R within infiltrating myeloid cells in brain (Braun et al. 2018). Mice treated with a selective CB2R agonist following CCI showed improved neurobehavioral outcomes associated with significant reduction in brain edema and various parameters of neuroinflammation (Song et al. 2022b).

The relationship between G-CSF treatment of TBI and mediation of repair via the eCBs may lead to greater understanding of brain repair and development of novel therapeutic agents. Administration of the Phyto cannabinoid Δ9-Tetrahydrocannabinol (THC) to mice after CCI was recently shown to enhance behavioral recovery associated with increased expression of G-CSF and other neurotrophic factors (Song et al. 2021). G-CSF treatment alone, after CCI, reversed the down-regulation of CB1R and the up-regulation of CB2R in cortex, hippocampus and striatum (Song et al. 2022b). Other researchers have reported that treatment with a CB2R agonist enhances brain repair and functional recovery after TBI (Shohami et al. 2011). Since THC and G-CSF can each enhance recovery from TBI, and THC treatment results in G-CSF upregulation, it was important to learn how G-CSF interacts with the endocannabinoid system following CCI. The hypothesis tested in the experiments described here is that CB2 receptors are critical in mediating the reparative effects of G-CSF after TBI. To test this, CBR2 knockout mice were used to determine if CB2R are required to mediate the reparative actions of G-CSF treatment.

## Methods

### Animals

The National Institutes of Health Guide for the care and use of laboratory animals was strictly followed. This research protocol was reviewed and approved by the institutional animal care and use committee of the University of South Florida. Adult, 3-month-old, male CB2-R knockout mice (B6.129P2-*Cnr2*^*tm1Dgen*^/J) were purchased from Jackson Laboratory (Bar Harbor, Maine). C57 BL/6 J, which served as normal control mice, were also purchased from Jackson Laboratory. Standard procedures were followed: after housing in animal facility cages, mice were left undisturbed for 1 week after arrival. Mice were provided free access to water and food. They were kept in a temperature- and humidity-controlled room on a 12-h light/12-h dark cycle with lights on at 7:00 AM. CB2-R KO mice (4 groups with *n* = 4 per group,) sustained CCI to the right frontal cortex. Group 1 = sham surgery, vehicle; Group 2 = sham surgery, G-CSF treatment; Group 3 = CCI, vehicle treatment; Group 4 = CCI, G-CSF treatment. Two groups of control mice (C57Bl/6 J) received vehicle or G-CSF (*n* = 6 per group). Mice were treated for 3 days after CCI with daily subcutaneous (s.c.) injections of G-CSF (100 microg/kg) or vehicle. Animals were euthanized on day 3 after CCI. Each brain was re-moved after perfusion with heparinized saline and dissected into three regions (cerebral cortex, corpus striatum, and hippocampus) for analyses.

### Motor balance and coordination measured with rotarod performance

The experimental setup involved using a rotarod apparatus (Model 47,600 rotarod for mice; Ugo Basile, Gemonio, Italy) to assess the motor balance and coordination of animals, with a focus on the effects of treatment (vehicle or G-CSF) following a controlled cortical impact (CCI) injury. The rotarod data, including training and testing trials, were collected at multiple time points to evaluate motor recovery in response to the treatments. Data were generated by averaging the scores (total time spent on the treadmill divided by three trials) for each animal during training and testing days. Each animal was placed in a neutral position on a cylinder, the rod was rotated, with the speed accelerated linearly from 4 to 40 rpm within 3 min, and the time spent on the rotarod was recorded automatically. For training, animals were given one trial before testing. For testing, animals were given three trials, and the average score on these three trials was used as the individual rotarod score. After baseline performance had been established, animals were randomly assigned to receive vehicle or G-CSF after administration of CCI. Rotometry was repeated on day 3 and day 7 after CCI.

### Surgery and CCI

Animals underwent an experimental TBI with an electromagnetic CCI device (Leica Biosystems, My NeuroLab.com), as described previously (Yu et al. 2009). Animals initially received meloxicam (5–10 mg/kg, s.c.) at the time of anesthesia induction (with 125 mg/kg ketamine and 12.5 mg/kg xylazine). After deep anesthesia had been achieved (verified by checking for pain reflexes), individual animals were fixed in a stereotaxic frame (David Kopf Instruments, Tujunga, CA). After exposing the skull, craniectomy (~ 3 mm to accommodate the impactor tip) was performed over the right frontoparietal cortex (0.5 mm anteroposterior and 0.5 mm mediolateral to bregma). All mice received a moderate TBI. The electromagnetic TBI device (with a convex tip diameter of 2.5 mm) impacts the brain at a velocity of 5.0 m/sec, reaching a depth of 1.0 mm below the dura mater layer, and re-mains in the brain for 150 ms. The impactor rod was angled at 158 deg. to the vertical to maintain a perpendicular position in reference to the tangential plane of the brain curvature at the impact surface. A contact sensor cable (Leica Biosystems) connected to the impactor measured velocity and duration to verify consistency. Bone wax was used to cover the craniectomized region, and the skin incision sutured thereafter. A computer-operated thermal blanket pad and a rectal thermometer allowed maintenance of body temperature within nor-mal limits. All animals were closely monitored until recovery from anesthesia and over the next 3 days.

### Drugs

G-CSF (Neupogen) was purchased from Amgen (Thousand Oaks, CA). The dose regimen of G-CSF (100 microg/kg) was chosen to replicate previous studies with this TBI model (Piomelli 2003).

### Quantitative real-time PCR of CB receptors

The mouse brain tissue samples were homogenized, and total RNA was purified using the RNeasy Mini Kit (Qiagen, Germantown, MD). One microgram of total RNA was used to synthesize the complementary cDNA with the iScript cDNA Synthesis Kit (Bio-Rad, Hercules, CA) according to the manufacturer’s protocol. The real-time PCR was performed using 2 microL of cDNA with 1 microL of 20 × TaqMan Mouse CB1-R primer/probe set (assay ID number Mm01212171_ s1) or CB2-R primer/probe set (assay ID number Mm02620087_s1) (Applied Biosystems, Foster City, CA), 10 microL of TaqMan Fast Advanced Master Mix (Applied Biosystems), and 7 microL of nuclease-free water for a total volume of 20 microL. As an internal control, a second real-time PCR was performed using 2 microL of cDNA with 1 microL 20 × TaqMan mouse GAPDH primer/probe set (Assay ID number Mm99999915_g1) (Applied Biosystems), 10 microL of 2 × TaqMan Fast Advanced Master Mix, and 7 microL of nuclease-free water for a total volume of 20 microL. Thermocycling conditions were as follows: 50 deg C, 2 min; 95 deg C, 10 min; and 40 cycles at 95 deg C for 15 s and 60 deg C for 1 min using the Applied BioSystems ViiA 7 System. Mouse CB1-R and CB2-R mRNA relative expression (RQ) to GAPDH was calculated. Each sample was run in duplicate.

### Mouse brain 2-AG enzyme-linked immunosorbent assay

Regions of brain were dissected, and tissue was processed according to the protocol for the enzyme-linked immunosorbent assay (ELISA) kit for mouse 2-AG (Cat# CE0443Ge; Cloud-Clone Corp. Katy, TX).

### Statistics

Data are expressed as mean – standard error of mean (*n* = 3–4 mice per data point). Statistical differences between parametric data were assessed using one-way analysis of variance (ANOVA) test with Bonferroni’s multi-group comparison, and non-parametric data were compared with the Kruskal–Wallis test with Dunn’s comparison (Prism 8.0, GraphPad). Sample sizes and number of replicates are included in the Figure legends or text. Survival was compared from all mice subjected to TBI using the Kaplan–Meier test. Differences between data were considered statistically significant when *p* < 0.05.

## Results

### Impact of GCSF treatment on behavioral outcomes

**Fig. 1:**
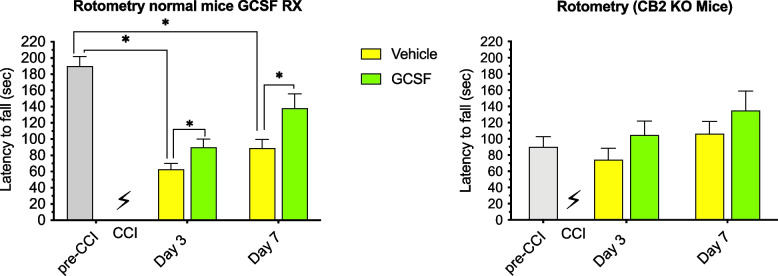
Baseline (pre-CCI) performance in normal control mice (left panel) was twice that of CB2 KO mice (right panel). Left panel: 2-way ANOVA (*n* = 4 mice per cohort) of control mice shows that both treatment and time contributed significantly to total variance (*p* < 0.05). Tukey’s multiple comparisons test showed G-CSF treatment enhanced recovery of rotometry performance on days 3 and 7 after CCI in normal mice (*p < 0.05). Right panel: 2-way ANOVA showed that neither treatment nor time had a significant effect on rotometry performance by CB2 KO (*n* = 4 mice per cohort) mice. Tukey’s multiple comparison testing did not reveal significant differences between vehicle and G-CSF treated mice

Compared to normal mice, CB2R KO mice exhibited different balance and coordination performances (measured by rotometry) before and after treatments with G-CSF. Before CCI, the latency to fall on the rotometer in control mice (190 s) was over twice as much as that in the CB2R KO mice (90 s) (Fig. 1). Non-transgenic control mice treated with vehicle after CCI exhibited a significant drop in performance on days 3 and 7 (Panel A, Fig. 1). 2-way ANOVA of control mice data showed that both treatment and time contributed significantly to total variance (*p* < 0.05).

G-CSF treatment significantly enhanced recovery of motor performance on both days 3 and 7 after CCI in normal mice (*p < 0.05). 2-way ANOVA of results from CB2R KO revealed that neither treatment nor time contributed significantly to total variance and G-CSF treatment did not improve performance compared to vehicle-treated mice (Panel B, Fig. 1).

### Expression of CB2R in the brain following CCI/GCSF treatment

**Fig. 2:**
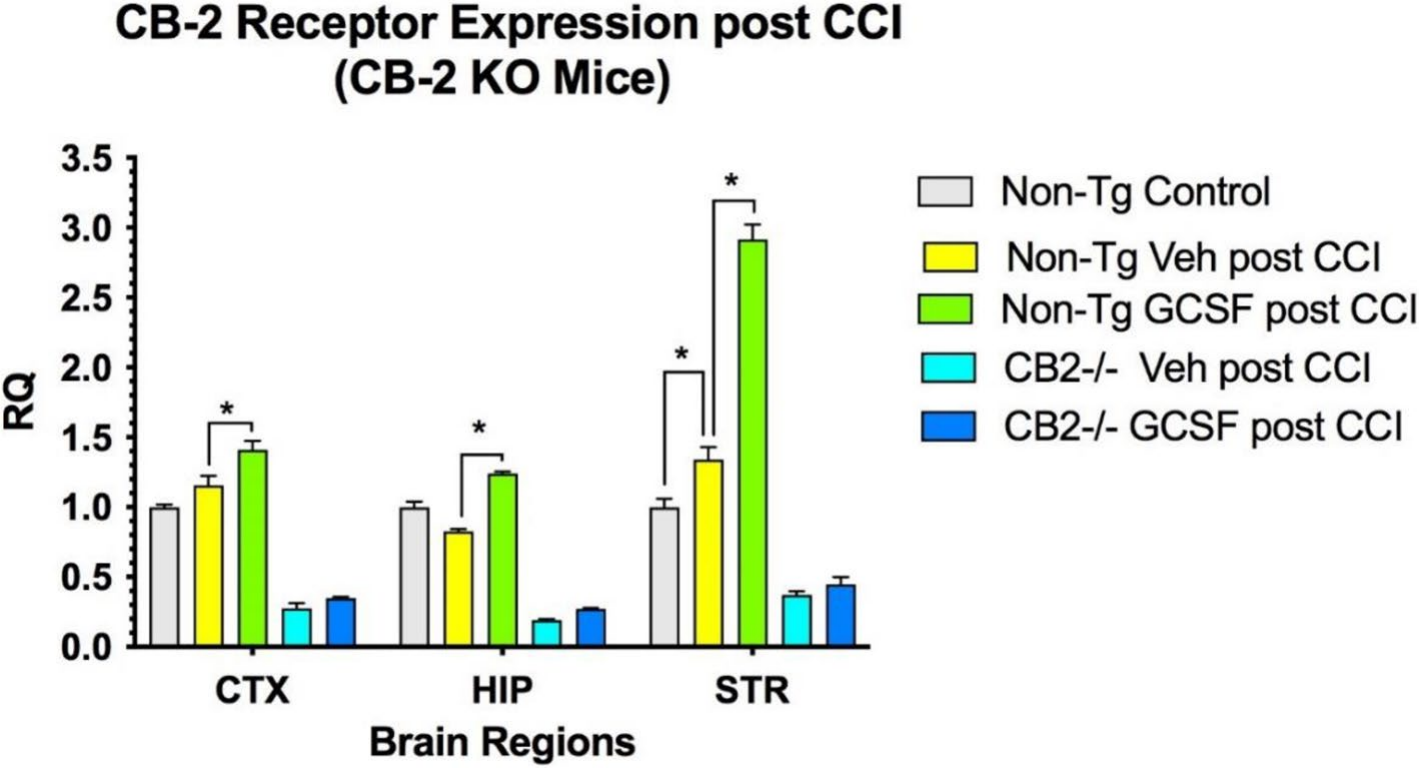
CB2R expression was increased only in normal controls. 2-way ANOVA indicates that treatment contributed 31% of total variance (*p* < 0.01) and brain region contributed 48% of total variance (*p* < 0.001). (*n* = 4 mice per cohort) Tukey’s multiple comparison tests show G-CSF treatment increased CB2 receptor expression significantly in the three brain regions in non-Tg control mice (*p* < 0.05) but not in In the CB2 knockout mice

**Fig. 3:**
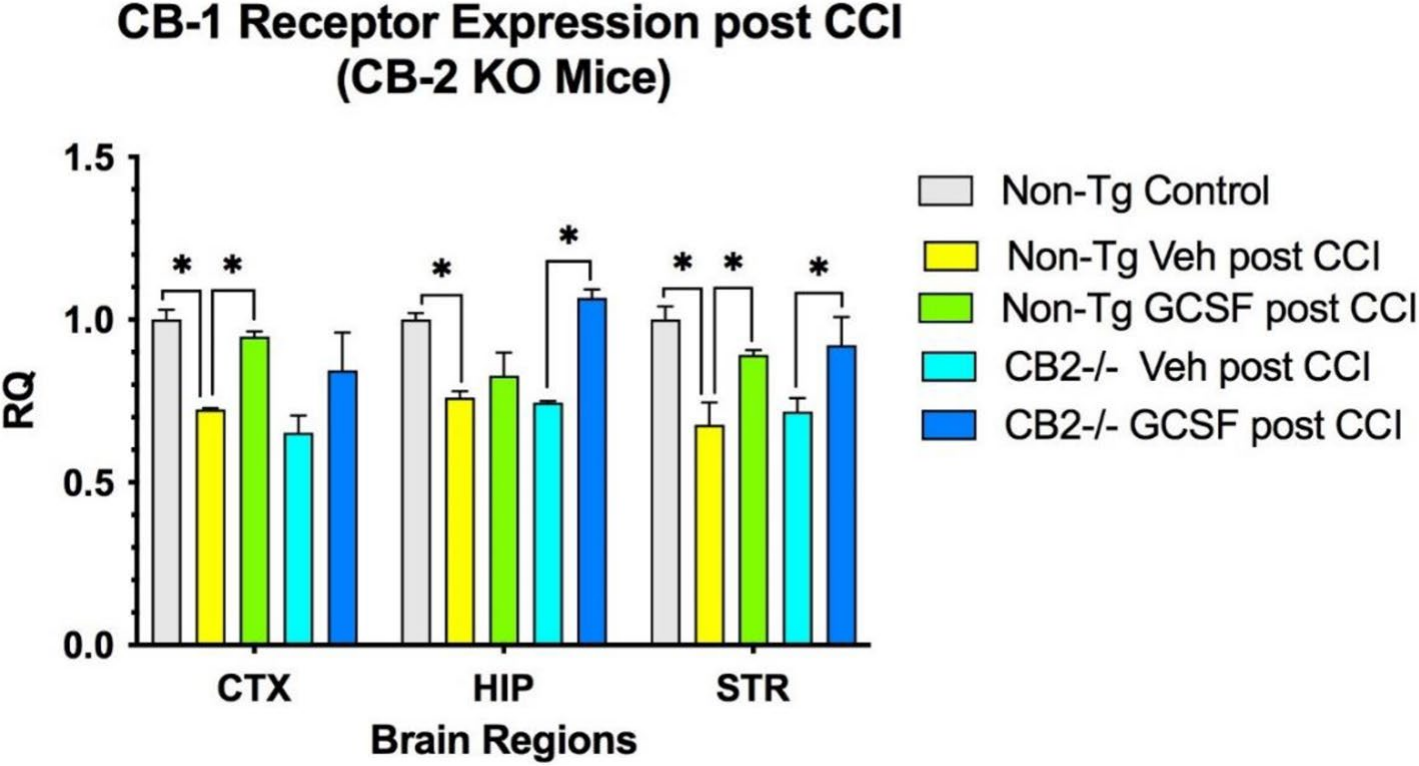
G-CSF treatment increased CB1 receptor expression significantly in CTX and STR in non-Tg control mice (*p < 0.05). (*n* = 4 mice per cohort) In the CB2 knockout mice, G-CSF treatment Increased expression of CB1 receptor significantly in HIP and STR (*p* < 0.05). 2-way ANOVA indicates that treatment contributed 51% of total variance (*p* < 0.01) and brain region contributed 15% of total variance (*p* = 0.09); this was followed by Sidak’s Multiple comparison tests showing significant differences between treatments (*p < 0.05)

CB2R expression in KO mice after CCI was detectable at low levels in CTX, HIP and STR, but did not change after G-CSF treatment (Fig. 2). Low level detection may be due to homology between the CB receptors in PCR analysis. In control mice, G-CSF treatment for 3 days following CCI significantly increased expression of CB2R in CTX, HIP and STR (*p* < 0.05) (Fig. 2). CB1R expression was reduced compared to baseline levels following CCI in both control and CB2R KO mice (Fig. 3). G-CSF treatment increased CB1 receptor expression significantly in CTX and STR (*p* < 0.05) in non-Tg control mice. In the CB2 knockout mice, G-CSF treatment increased expression of CB1 receptor significantly in HIP and STR (*p* < 0.05).

### Expression of GDNF, BDNF and 2-AG in the brain following CCI/GCSF treatment

**Fig. 4:**
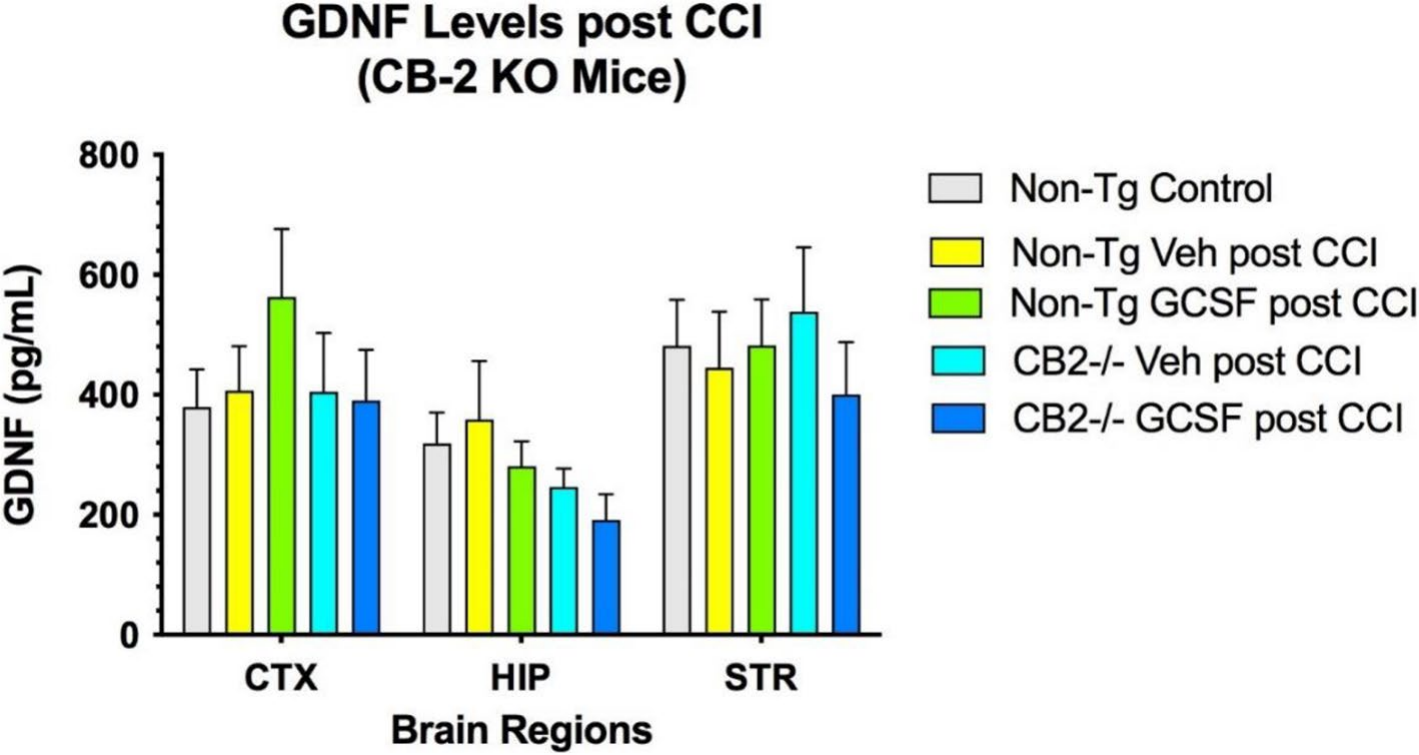
CGSF treatment did not alter GDNF levels in control or CB2R KO mice in the 3 brain regions. 2-way ANOVA showed that brain regions contributed 29% of total variation (*p* < 0.05) and treatment effect contributed 6% to total variation. (*n* = 4 mice per cohort) Tukey’s multiple comparisons test did not reveal significant differences in GDNF levels between treatments

**Fig. 5:**
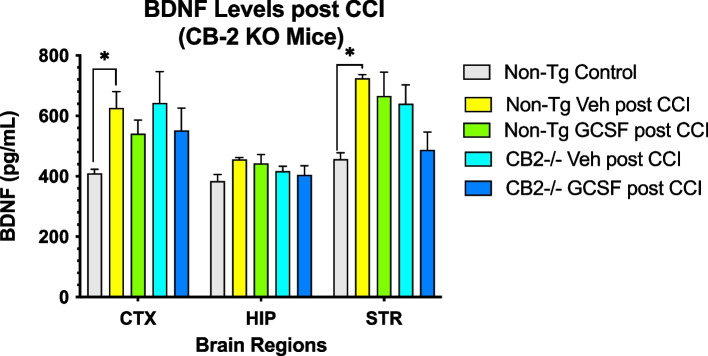
CCI resulted in increased BDNF levels in cortex and striatum (* = *p* < 0.05). 2-Way ANOVA revealed brain regions accounted for 37.5% of total variance and treatment accounted for 10.2% of total variance. (*n* = 4 mice per cohort). Tukey's multiple comparisons did not reveal differences between treatments with G-CSF and vehicle in both controls and CB2R KO mice

**Fig. 6:**
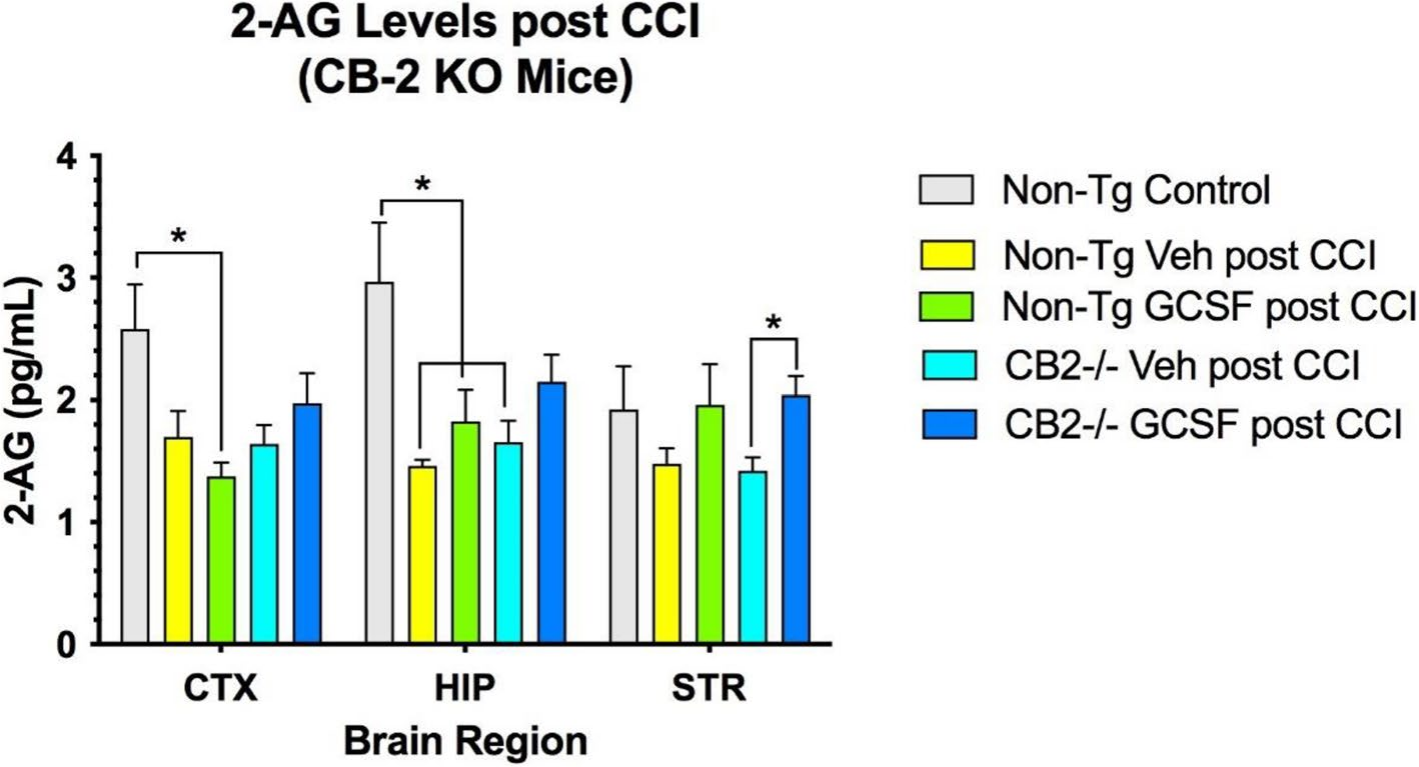
Levels of 2-AG were decreased by CCI in CTX and HIP. In STR 2-AG levels were significantly increased by G-CSF treatment, compared to vehicle treatment (*p < 0.05). 2-way ANOVA showed that treatment effect contributed 41% of total variation (*p* < 0.05). Brain regions contributed 3% of total variation. (*n* = 4 mice per cohort). Tukey's multiple comparisons revealed significant differences between controls and post-CCI levels of 2-AG in CTX and HIP and significant differences between treatments with G-CSF and vehicle in STR of CB2R KO mice (* = *p* < 0.05)

Levels of the neurotrophic factor GDNF, BNDF and 2-AG in 3 brain regions was not changed by G-CSF administration after CCI in both control and CB2R KO mice (Fig. 4). In contrast, the levels of BDNF increased in the cortex and striatum compared to both control and CB2R KO mice (Fig. 5). Finally, the levels of 2-AG were decreased by CCI in CTX and HIP. However, in the STR 2-AG levels were significantly increased by G-CSF treatment, compared to vehicle treatment (Fig. 6).

## Discussion

Recent work from our laboratory has shown that G-CSF treatment of mice after CCI resulted in enhanced brain repair, increased neurogenesis and improved performance in a radial arm water maze (Song et al. 2016). In addition, administration of the Phyto cannabinoid Δ9-Tetrahydrocannabinol (THC) to mice after CCI was recently shown to enhance behavioral recovery associated with increased expression of G-CSF and other neurotrophic factors (Song et al. 2021). Alterations in the eCBs (down-regulation of CB1 receptors and the upregulation of the CB2 receptors) triggered by CCI was reversed by G-CSF treatment. Since THC and G-CSF can each enhance recovery from TBI, and THC treatment results in G-CSF upregulation, it was important to learn how G-CSF interacts with the endocannabinoid system following CCI. This was tested by administration of cannabinoid (CB) receptor antagonists targeting CB1 and CB2 receptors. Pharmacological blockade of CB1 and CB2 receptors did not prevent the beneficial actions of G-CSF in promoting recovery (Braun et al. 2018). The results must be interpreted with caution since the outcome was based on the use of single doses of CB receptor drugs, without exploration of higher and lower doses. Moreover, CB receptor antagonists, especially at higher doses, can interact with other CB and non-CB receptors. To eliminate the problem of nonspecific interaction of CB receptor antagonists with other receptors, these experiments were repeated using mice in which the CB2 receptor is “knocked out”.

CB2 receptors are primarily expressed in peripheral cells and are associated with the immune system. However, it is now recognized that CB2 receptors are also found in the CNS. They are expressed by CNS resident inflammatory cells, microglial cells, dendritic cells (Yu et al. 2009; Maresz et al. 2005), and brain endothelial cells (Pertwee 2008). Activation of CB2 receptors has been reported to mediate brain repair and improve behavioral recovery in a mouse model of TBI by various mechanisms including recruitment of peripheral monocytes to the site of injury in the brain, promotion of anti-inflammatory cytokine production and improving blood flow to the site of injury (Song et al. 2022b).

Using CB2R KO mice, we demonstrated that G-CSF treatment did not enhance recovery of balance and coordination measured on the rotometer, unlike the beneficial effects of G-CSF treatment observed in normal control mice. Expression of CB2R, which is increased by CCI, was further increased by G-CSF treatment in normal mice 3 days after CCI. In contrast, CB1R expression was down-regulated following CCI in normal mice and G-CSF treatment significantly reversed this effect, confirming an earlier report (Braun et al. 2018). In addition, the CB1R in the CB2R KO mice was also down-regulated by the trauma and reversed by G-CSF treatment indicating that “knocking-out” or lowering expression of CB2R did not impact expression of CB1R. It should be noted that the CB2R KO mice did not have complete ablation of the receptor; it was still measurable in all the brain regions of these KO mice.

The primary interpretation of these data is that the CB2R must be active for enhancement of recovery of balance and coordination following CCI. In addition, the CB2R must be active for mice to exhibit normal balance and coordination, as measured on the rotometer. These data are in conflict with our earlier report that pharmacological blockade of the CB2R did not prevent the beneficial reparative responses to G-CSF treatment (Braun et al. 2018; Golech et al. 2004). The earlier conclusion that CB receptors were not required to mediate recovery from CCI included several caveats: a) results were based on the use of single doses of CBR antagonists, without exploration of higher and lower doses. Moreover, CBR antagonists, especially at higher doses, can interact with other CB and non-CB receptors.

This new data obtained from a CB2R deficient mouse supports the hypothesis that activation of these endocannabinoid receptors is critical in mediating the reparative effects of G-CSF treatment. Without the CB2R, the downstream effects of G-CSF treatment that appear to play a role in enhanced recovery from CCI in normal mice appears to be deficient as well. This includes expression of the neurotrophic factors BDNF and GDNF, anti-inflammatory cytokines and levels of the endogenous CB ligand 2-AG, all of which are involved in the reparative process promoted by G-CSF (Song et al. 2016). In CB2R KO mice, G-CSF treatment did not change or increase levels of BDNF and GDNF in any of the brain regions. Levels of the most abundant cannabinoid ligand (2-AG) were shown to increase following G-CSF treatment in the CB2R KO mice, and yet this did not translate into enhanced recovery of locomotor activity for reasons that need to be explored going forward. In particular, examination of molecular, biochemical, cellular and behavioral changes will require expansion of the time scale for recovery from hours after CCI to 14 days after the injury.

In summary, we demonstrated that G-CSF administration for 3 days after CCI did not enhance recovery of balance and coordination measured on the rotometer in CB2R KO mice, unlike the beneficial effects of G-CSF treatment observed in normal control mice. Even before CCI, the CB2R mice were markedly impaired on the rotometer, suggesting that activity of CB2R is important for normal function of neural networks that mediate balance and coordination. Expression of CB2R was increased by G-CSF treatment in normal mice 3 days after CCI but not in CB2R KO mice. Interestingly, the CB1R in the CB2R KO mice was upregulated by G-CSF treatment indicating that “knocking-out” or lowering expression of CB2R did not impact expression of CB1R. Expression of the neurotrophic factors BDNF and GDNF were not changed by G-CSF treatment in CB2R KO mice. Levels of the endogenous cannabinoid ligand, 2-AG were shown to be increased by G-CSF treatment in the CB2R KO mice, but this effect was insufficient to promote recovery of balance and coordination.

## Data Availability

The data that support the findings of this study are not openly available due to reasons of sensitivity and are available from the corresponding author upon reasonable request. Data are located in controlled access data storage at the Haley VA.

## Abbreviations

G-CSF: Granulocyte-Colony Stimulating Factor
eCS: Endocannabinoid system
CB: Cannabinoid
BDNF: Brain-derived neurotrophic factor
HGF: Hematopoietic growth factor
2-AG: 2-Arachidonoyl-glycerol
CB1: Cannabinoid-1 receptor
CB2R: Cannabinoid-2 receptor

## References

[CR1] Braun M, Khan ZT, Khan MB, Kumar M, Ward A, Achyut BR, Arbab AS, Hess DC, Hoda MN, Baban B, et al. Selective activation of cannabinoid receptor-2 reduces neuroinflammation after traumatic brain injury via alternative macrophage polarization. Brain Behav Immun. 2018;68:224–37. 10.1016/j.bbi.2017.10.021. 29079445 10.1016/j.bbi.2017.10.021 PMC5767553

[CR2] Cernak I. Animal models of head trauma. NeuroRx. 2005;2:410–22. 16389305 10.1602/neurorx.2.3.410 PMC1144485

[CR3] Chen C. Inhibiting degradation of 2-arachidonoylglycerol as a therapeutic strategy for neurodegenerative diseases. Pharmacol Ther. 2023;244: 108394. 10.1016/j.pharmthera.2023.108394. 36966972 10.1016/j.pharmthera.2023.108394 PMC10123871

[CR4] Golech SA, McCarron RM, Chen Y, Bembry J, Lenz F, Mechoulam R, Shohami E, Spatz M. Human brain endothelium: coexpression and function of vanilloid and endocannabinoid receptors. Brain Res Mol Brain Res. 2004;132:87–92. 10.1016/j.molbrainres.2004.08.025. 15548432 10.1016/j.molbrainres.2004.08.025

[CR5] Lopez-Rodriguez AB, Acaz-Fonseca E, Viveros MP, Garcia-Segura LM. Changes in cannabinoid receptors, aquaporin 4 and vimentin expression after traumatic brain injury in adolescent male mice. Association with edema and neurological deficit. PloS one. 2015;10:e0128782. 10.1371/journal.pone.0128782. 10.1371/journal.pone.0128782 PMC4454518 26039099

[CR6] Maresz K, Carrier EJ, Ponomarev ED, Hillard CJ, Dittel BN. Modulation of the cannabinoid CB2 receptor in microglial cells in response to inflammatory stimuli. J Neurochem. 2005;95:437–45. 10.1111/j.1471-4159.2005.03380.x. 16086683 10.1111/j.1471-4159.2005.03380.x

[CR7] Pertwee RG. Ligands that target cannabinoid receptors in the brain: from THC to anandamide and beyond. Addict Biol. 2008;13:147–59. 10.1111/j.1369-1600.2008.00108.x. 18482430 10.1111/j.1369-1600.2008.00108.x

[CR8] Piomelli D. The molecular logic of endocannabinoid signalling. Nat Rev Neurosci. 2003;4:873–84. 10.1038/nrn1247. 14595399 10.1038/nrn1247

[CR9] Schabitz WR, Kollmar R, Schwaninger M, Juettler E, Bardutzky J, Scholzke MN, Sommer C, Schwab S. Neuroprotective effect of granulocyte colony-stimulating factor after focal cerebral ischemia. Stroke. 2003;34:745–51. 12624302 10.1161/01.STR.0000057814.70180.17

[CR10] Schurman LD, Lichtman AH. Endocannabinoids: a promising impact for traumatic brain injury. Front Pharmacol. 2017;8:69. 10.3389/fphar.2017.00069. 28261100 10.3389/fphar.2017.00069 PMC5314139

[CR11] Shohami E, Cohen-Yeshurun A, Magid L, Algali M, Mechoulam R. Endocannabinoids and traumatic brain injury. Br J Pharmacol. 2011;163:1402–10. 10.1111/j.1476-5381.2011.01343.x. 21418185 10.1111/j.1476-5381.2011.01343.x PMC3165950

[CR12] Shyu WC, Lin SZ, Lee CC, Liu DD, Li H. Granulocyte colony-stimulating factor for acute ischemic stroke: a randomized controlled trial. CMAJ. 2006;174:927–33. 16517764 10.1503/cmaj.051322 PMC1405861

[CR13] Six I, Gasan G, Mura E, Bordet R. Beneficial effect of pharmacological mobilization of bone marrow in experimental cerebral ischemia. Eur J Pharmacol. 2003;458:327–8. 12504790 10.1016/s0014-2999(02)02785-1

[CR14] Solaroglu I, Cahill J, Jadhav V, Zhang JH. A novel neuroprotectant granulocyte-colony stimulating factor. Stroke. 2006;37:1123–8. 16514095 10.1161/01.STR.0000208205.26253.96

[CR15] Song S, Kong X, Acosta S, Sava V, Borlongan C, Sanchez-Ramos J. Granulocyte colony-stimulating factor promotes behavioral recovery in a mouse model of traumatic brain injury. J Neurosci Res. 2016;94:409–23. 10.1002/jnr.23714. 26822127 10.1002/jnr.23714 PMC4805459

[CR16] Song S, Kong X, Borlongan C, Sava V, Sanchez-Ramos J. Granulocyte colony-stimulating factor enhances brain repair following traumatic brain injury without requiring activation of cannabinoid receptors. Cannabis Cannabinoid Res. 2021;6:48–57. 10.1089/can.2019.0090. 33614952 10.1089/can.2019.0090 PMC7891202

[CR17] Song S, Kong X, Wang B, Sanchez-Ramos J. Administration of Δ^9^-tetrahydrocannabinol following controlled cortical impact restores hippocampal-dependent working memory and locomotor function. Cannabis Cannabinoid Res. 2022a;7(4):424–35. 10.1089/can.2021.0053. 34747647 10.1089/can.2021.0053 PMC9418466

[CR18] Song S, Kong X, Wang B, Sanchez-Ramos J. Recovery from traumatic brain injury following treatment with Δ9-tetrahydrocannabinol is associated with increased expression of granulocyte-colony stimulating factor and other neurotrophic factors. Cannabis Cannabinoid Res. 2022b;7(4):415–23. 10.1089/can.2020.0119. 33998887 10.1089/can.2020.0119 PMC9418356

[CR19] Yang DY, Chen YJ, Wang MF, Pan HC, Chen SY, Cheng FC. Granulocyte colony-stimulating factor enhances cellular proliferation and motor function recovery on rats subjected to traumatic brain injury. Neurol Res. 2010;32:1041–9. 20810026 10.1179/016164110X12807570510013

[CR20] Yu S, Kaneko Y, Bae E, Stahl CE, Wang Y, van Loveren H, Sanberg PR, Borlongan CV. Severity of controlled cortical impact traumatic brain injury in rats and mice dictates degree of behavioral deficits. Brain Res. 2009;1287:157–63. 19573519 10.1016/j.brainres.2009.06.067

[CR21] Zhu D, Gao F, Chen C. Endocannabinoid metabolism and traumatic brain injury. Cells. 2021;10(11):2979. 10.3390/cells10112979. 34831202 10.3390/cells10112979 PMC8616221

